# A CRITICAL CARE PATHWAY BASED ON ICOPE TO PROMOTE HEALTHY AGING OF OLDER ADULTS

**DOI:** 10.1093/geroni/igae098.0543

**Published:** 2024-12-31

**Authors:** Doris Sau Fung Yu, Yiting Tang, Shuangzhou Chen, Feng Chen

**Affiliations:** The University of Hong Kong, Hong Kong, China (People’s Republic); The University of Hong Kong, Hong Kong, China (People’s Republic); The University of Hong Kong, Hong Kong, China (People’s Republic); The University of Hong Kong, Hong Kong, China (People’s Republic)

## Abstract

The United Nations Decade of Healthy Aging 2021-2030 calls for proactive and collaborative strategies to optimize the functional capacity of older adults. Aligning with the World Health Organization advocacy of optimizing the functional capacity of older adults through increasing the compatibility of their intrinsic capacity (IC) of older adults and the environment, the research team has developed the 12-week Pathway to Healthy Aging to translate the ICOPE care model to the Chinese community. The 12-week Pathway to Healthy Aging is a health-social collaborative model that adopts the WHO-ICOPE approach, together with the concepts of clinical critical care pathway, case management, empowerment-based behavioral modification and health-oriented peer coaching to promote healthy aging to the older adults with signs of accelerated aging. From September 2022 to June 2023, the care model was provided to 806 targeted clients recruited from a territory-wide region in Hong Kong. The speaker will share experiences and challenges of translating the WHO ICOPE care model in a Chinese community. More specifically, she will share the insights on: i) how to integrate the ICOPE assessment data to identify the health care priority of older adults, ii) method to develop the critical care pathway to promote person-center care and longer-term behavioral change for healthy aging promotion, iii) strategies to optimize effective collaboration between the nurses and health-oriented peer coach in case management. The presentation will conclude with remarks to inform future initiatives aimed at preparing society for an aging population.

